# Nonsmall-cell lung cancer treatment: current status of drug repurposing and nanoparticle-based drug delivery systems

**DOI:** 10.55730/1300-0152.2687

**Published:** 2024-04-03

**Authors:** Tuğba Gül İNCİ, Serap ACAR, Dilek TURGUT-BALIK

**Affiliations:** Department of Bioengineering, Faculty of Chemical and Metallurgical Engineering, Yıldız Technical University, İstanbul, Turkiye

**Keywords:** Drug repurposing, nonsmall-cell lung cancer, NSCLC, nanoparticle-based drug delivery systems

## Abstract

Drug repurposing is the strategy of drug utilization for a treatment option other than the intended indications. This strategy has witnessed increased adoption over the past decades, especially within cancer nanomedicine. Cancer nanomedicine has been facilitated through nanoparticle-based (NP-based) delivery systems which can combat nonsmall-cell lung cancer (NSCLC) via recent advances in nanotechnology and apply its benefits to existing drugs. The repurposing of drugs, coupled with NP-based drug delivery systems, presents a promising avenue for achieving effective therapeutic solutions with accelerated outcomes. This review aims to present an overview of NSCLC treatments, with a specific focus on drug repurposing. It seeks to elucidate the latest advances in clinical studies and the utilization of NP-based drug delivery systems tailored for NSCLC treatment. First, the molecular mechanisms of Food and Drug Administration (FDA)-approved drugs for NSCLC, including ROS1 tyrosine kinase inhibitors (TKI) like repotrectinib, approved in November 2023, are detailed. Further, in vitro studies employing a combination strategy of drug repurposing and NP-based drug delivery systems as a treatment approach against NSCLC are listed. It includes the latest study on nanoparticle-based drug delivery systems loaded with repurposed drugs.

## 1. Introduction

Drug repurposing is the strategy of modifying the utilization of approved drugs for treatment options beyond their originally intended indications (Ashburn and Thor, 2004). This strategy accelerates time-consuming and costly drug development process by reducing pharmacokinetic uncertainties (Ashburn and Thor, 2004). Therefore, repurposing attracts attention in the pharmaceutical industry for diseases like cancer, infectious diseases, orphan illnesses, and neglected diseases (Pushpakom et al., 2019; Juárez López and Schcolnik Cabrera, 2021; Pantziarka et al., 2021). The repurposing strategy emerged following a failed clinical trial, marking one of its earliest applications (Raje and Anderson, 1999). Thalidomide, originally approved for treating morning sickness, failed and resulted in a tragic incidence of malformed infants. Subsequently, it was first repurposed for erythema nodosum leprosum and then for cancer, particularly in cases of newly diagnosed multiple myeloma (Raje and Anderson, 1999). Since then, repurposing has emerged as a strategy to address unmet therapeutic needs in cancer treatment (World Health Organization, 2021). Repurposing approaches involve the identification of target molecules not only for cancer and but also for a broad range of diseases, employing both experimental and computational methodologies (Pushpakom et al., 2019; Dalwadi et al., 2023). In computational approaches, algorithms, genomics, evidence, pathways, toxicity assessments, as well as drug- and target-based applications are utilized. On the other hand, experimental approaches employ binding assays to identify target interactions and phenotypic screening, both of which contribute to enhancing the discovery of repurposed drugs (Pushpakom et al., 2019; Dalwadi et al., 2023).

According to estimates from the International Agency for Research on Cancer, there were approximately 19.3 million new cancer cases and around 10 million deaths attributed to cancer in 2020 (Sung et al., 2021). Among all cancer types, lung cancer exhibited the highest mortality rate, accounting for an estimated 1.8 million deaths globally in 2020 (Sung et al., 2021). It caused 13.7% and 21.5% of the total female and male cancer deaths, respectively, making it the most prevalent cancer for males with 14.3% (Sung et al., 2021). While breast cancer had the highest incidence among females at 24.5%, followed by colorectal cancer at 9.4%, lung cancer ranked third with a percentage of 8.4% (Sung et al., 2021).

Lung cancers are diagnosed pathologically with the classification of several different tumors (Nicholson et al., 2022). World Health Organization classification of lung tumors, initially based on immunohistochemistry, was updated in 2021 to rely on molecular pathology (Nicholson et al., 2022). Thoracic tumors are classified into subtypes including papillomas, adenomas, adenocarcinomas, squamous cell carcinomas, other epithelial tumors, salivary gland-type tumors, lung neuroendocrine neoplasms, neuroendocrine tumors, neuroendocrine carcinomas, tumors of ectopic tissues, lung-specific mesenchymal tumors, PEComatous tumors, and hematolymphoid tumors (Nicholson et al., 2022). Lung cancer is mainly divided into two main groups: small cell lung cancer (SCLC) and nonsmall-cell lung cancer (NSCLC), NSCLC representing about 80% of all lung cancers (Wakelee et al., 2007; Subramanian et al., 2013; Thai et al., 2021; American Cancer Society, 2023).

Treatment options for NSCLC are surgery, radiation, chemotherapy, targeted treatments, and immunotherapy alone or in combination (Thai et al., 2021). These options are evolving thanks to advancements in screening technologies, a deeper understanding of disease mechanisms, and ongoing investigations into the effectiveness of existing therapies. Examples include immune checkpoint inhibitor therapy for advanced NSCLC patients, lobectomy for early-stage NSCLC patients, and postoperative radiotherapy for unresectable stage III NSCLC patients (Mouritzen et al., 2021; Thai et al., 2021). Understanding disease mechanisms has facilitated the design of target-specific drugs with the use of nanotechnological systems (Fan et al., 2023a). Recent advances in NP-based drug delivery systems leverage targeted drug delivery to overcome obstacles like systemic distribution and drug side effects (Dang and Guan, 2020; Fan et al., 2023a).

**Figure 1:**
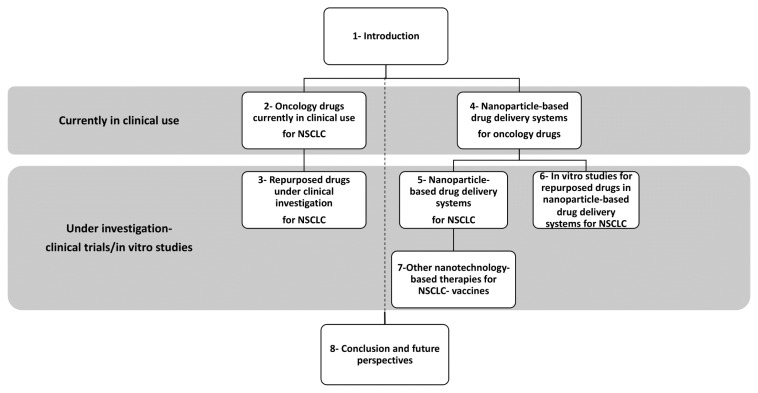
Structure of the review.

This review provides an overview of repurposed NSCLC drugs, along with currently used NSCLC drugs, and NP-based drug delivery systems tailored for NSCLC (Figure 1).

## 2. Oncology drugs currently in clinical use for NSCLC

**Figure 2:**
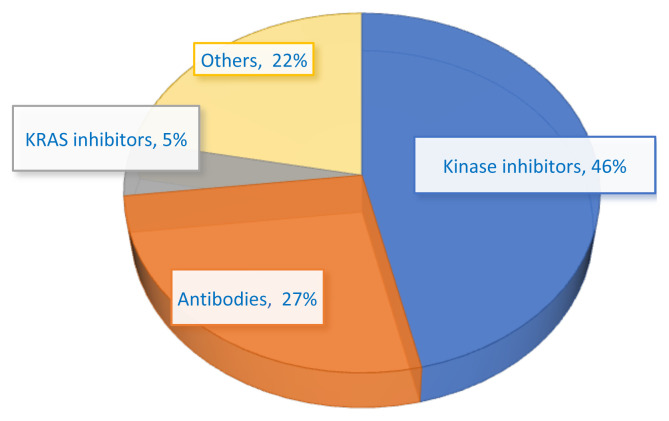
Percentage of the approved active substances for NSCLC drugs (NIH, 2023)1

To date, a total of 39 drugs have been approved for the treatment of NSCLC to date (Table 1) (NIH, 2023)^1^. These drugs are predominantly tyrosine kinase inhibitors (TKIs), including anaplastic lymphoma kinase (ALK) inhibitors, epidermal growth factor receptor (EGFR) inhibitors, MET receptor TKIs, and rearranged during transfection (RET) receptor TKIs. Additionally, a few antibodies and Kirsten rat sarcoma (KRAS) inhibitors are used in NSCLC treatment (Figure 2) (Wishart et al., 2018; NIH, 2023^1^). In addition to the NIH NSCLC drug list, updated as of October 23rd, 2023^1^, the first c-ROS oncogene 1 (ROS1) TKI for NSCLC named repotrectinib was approved on November 15th, 2023^2^ (Table 1).

TKIs are main molecules of targeted therapy against pathways related to the regulation of cell growth in NSCLC via binding of receptor tyrosine kinase. Among 19 receptor kinase families identified in the human genome (Robinson et al., 2000), ALK, EGFR, MET, and RET tyrosine kinases have become targets for the treatment of NSCLC (Broekman et al., 2011). This is due to the presence of oncogenic driver mutations for NSCLC occurring on ALK, EGFR, ROS1, V-raf murine sarcoma viral oncogene homolog B (BRAF), (MET, NTRK, RET, KRAS, Human epidermal growth factor 2 (HER2) and NRG1 (Chevallier et al., 2021). ALK-TKIs were discovered through the molecular characterization of NSCLC, specifically with the identification of the *EML4-ALK* fusion gene (Soda et al., 2007). Crizotinib was the first ALK-TKI approved by the FDA in 2011, leading to a shift in first-line therapy option was changed from pemetrexed-plus-platinum to crizotinib for the *ALK*-positive NSCLC (Malik et al., 2014; Solomon et al., 2014). The drug targets ALK, ROS-1, and c-MET but causes resistance in long-term administration by bypassing the blood-brain barrier leading to poor brain penetration (Costa et al., 2011; Casaluce et al., 2016). Following the discovery of crizotinib, other ALK-TKIs have been developed, including ceritinib, alectinib, brigatinib (AP26113), and lorlatinib (Testa et al., 2023). While these ALK-TKIs overcome the brain penetration problem, ALK-TKI resistance mechanisms remain a challenge (Testa et al., 2023) even for the third-generation ALK-TKI lorlatinib which acts as an ATP-competitive small molecule inhibitor (Syed, 2019). Repotrectinib, the most recently approved TKI, received approval in November 2023. It specifically targets ROS1-positive NSCLC patients (FDA, 2023)^2^. It is a TRIDENT-1 trial (NCT03093116) drug and showed an overall response rate of 14.8 (7.6–NE) months and a progression-free survival of 9.0 (6.8–19.7) months among ROS-1-positive NSCLC patients previously treated with ROS1 TKI and platinum-based chemotherapy (Cho et al., 2023). In the trial, the ROS1 TKI-naïve group and the group previously treated with a ROS1 inhibitor demonstrated a verified overall response rate of 79% (95% CI: 68, 88) and 38% ORR (95% CI: 25, 52), respectively (FDA, 2023)^2^.

EGFR-TKIs mostly target the allosteric and ATP sites of EGFR, exerting ATP-compatible and/or irreversible action (Singh et al., 2023). EGFR mutations guide the EGFR-TKI development starting from Del19/L858R mutation to T79M (Singh et al., 2023). In 2003, gefitinib was approved by the FDA as the first EGFR-TKI for treating NSCLC, specifically targeting the Del19/L858R mutation (Cohen et al., 2004). The most recent FDA-approved EGFR-TKI for NSCLC is mobocertinib, which targets EGFR exon 20 insertion mutation (Markham, 2021). Osimertinib, a third-generation EGFR-TKI, targets the T79M mutation in patients sensitized to EGFR or those with resistance-associated mutations. However, resistance to osimertinib has also been reported (Gomatou et al., 2023). One potential approach to address EGFR drug resistance could involve therapies using antibodies against C797S mutation, which remains a challenge (Singh et al., 2023). Necitumumab is an example of an established EGFR monoclonal antibody for NSCLC. It blocks the ligand by binding to the extracellular domain III of EGFR (Dienstmann and Tabernero, 2010; Cai et al., 2020; NIH, 2023^1^). In addition to EGFR antibodies, other antibodies developed and approved for NSCLC are listed in Table 1 (NIH, 2023)^1^. A broad range of antibodies, ranging from IgG monoclonal antibodies to antibody derivatives, antibody-drug conjugates, and immunocytokines bolster immune system functions by inhibiting cancer cell activity and eradicating cancer cells (Jin et al., 2022). Programmed death ligand 1 (PD-L1)-targeting antibodies, such as nivolumab and ipilimumab, were found to be effective for NSCLC. However, the need for further biomarkers for effective treatment has been highlighted (Hellmann et al., 2019; Chiang and Herbst, 2020).

KRAS mutations have been found in 20%–25% of NSCLC patients, with the G12C mutation specifically identified as a drug target (Friedlaender et al., 2020). The FDA recently approved two KRAS (G12C) inhibitors, sotorasib (AMG-510) in 2021 and adagrasib (MRTX849) in 2022 (Blair, 2021; Dhillon, 2023). These drugs are irreversible inhibitors that covalently bind to G12C, thereby locking inactive state KRAS (Blair, 2021; Dhillon, 2023). However, further improvements are needed for these inhibitors as they are associated with intrinsic resistance, and related biomarkers are not available yet (Huang et al., 2021).

Despite extensive utilization of TKIs in nonresistant cases, they are associated with numerous side effects, including cardiovascular side effects such as arrhythmia, QT prolongation, bradycardia, hypertension, myocardial infarction, PR interval prolongation, atrioventricular block, left ventricular dysfunction, edema, heart failure, arrhythmia, and pericardial effusion (Shyam Sunder et al., 2023). Additionally, new TKIs or other anticancer drugs are needed because of growing resistance to existing TKIs (Ferguson and Gray, 2018; Wang and Wang, 2021). A first-generation ALK-TKI crizotinib and a second-generation ALK-TKI ceritinib were reported to cause bradycardia as an adverse effect (Wang and Wang, 2021). Currently, an FDA- approved third-generation ALK-TKI, lorlatinib, is considered the gold standard for ALK-TKI treatment. Additionally, fourth-generation ALK-TKIs have started their phase studies. However, more molecular characterization is needed for new-generation ALK-TKIs to overcome resistance (Syed, 2019; Testa et al., 2023).

Despite the availability of active substances for the treatment of NSCLC, mortality rates remain for the disease due to several reasons, such as on-target and off-target resistance to TKIs and the absence of related biomarkers (De Mello et al., 2020; Mustachio and Roszik, 2020; Gomatou et al., 2023; Singh et al., 2023; Testa et al., 2023). As drug development for NSCLC is challenging, new therapeutic options are needed (De Mello et al., 2020; Gomatou et al., 2023; Testa et al., 2023). Drug repurposing could expedite the drug discovery process, offering a solution to the global burden of cancer, including NSCLC (Zhang et al., 2020; World Health Organization, 2021).

## 3. Repurposed drugs under clinical investigation for NSCLC

Drug repurposing in cancer provides a way to overcome drug development challenges, including low success rate of clinical studies, life-threatening side effects of approved drugs, and rapidly developed resistance mechanisms to approved drugs (Sleire et al., 2017). Anticancer drug development has the lowest approval rate after phase 1 (3.4%) compared to other drug development areas such as cardiovascular (25.5%), infectious (25.2%), and autoimmune (15.1%) diseases (Wong et al., 2019). Since drug repurposing eliminates pharmacokinetic uncertainty, anticancer drug development can benefit from this strategy (Sleire et al., 2017).

Repurposed drugs for NSCLC are currently undergoing clinical investigations. Studies exploring the treatment of NSCLC with repurposed drugs like nonsteroidal antiinflammatory drugs (NSAIDs), steroids, protease inhibitors, statins, antihyperglycemics, β-Blockers, antifungals, and antivirals have been conducted (clinicaltrials.gov)^6^. Up-to-date clinical trials on repurposed drugs for NSCLC are presented in Table 2, while repurposed active substances from FDA orphan drugs and non-FDA-approved substances that are undergoing clinical trials for NSCLC are listed in Table 3^6^. Drugs listed in Table 2 are undergoing clinical trials for several purposes like decreasing morbidity, determining their potential efficacy after gaining a more detailed understanding of their biological effects, and investigating their ability to meet the need for new drugs due to resistance to existing medications in the NSCLC mechanism.

A life-threatening side effect of conventional NSCLC radiotherapy is cardiac morbidity, which may result in higher mortality for the patients (Donovan et al., 2023). An interventional phase 3 study (NCT04980716) conducted with NSCLC patients at risk of cardiac events after chemoradiotherapy utilizes statins to lower cardiac morbidity. This study, initiated in 2021, is currently in the recruitment phase and is expected to conclude in July 2026 (NCT04980716).

Another morbidity, venous thromboembolism (VTE), is associated with cancer, necessitating thromboprophylaxis after cancer progression (Weitz et al., 2020). A phase 3 clinical trial conducted between 2013 and 2016 investigated the efficacy and safety of nadroparin for thromboprophylaxis. However, the results of this trial have not been posted (NCT01980849).

Thromboprophylaxis was recommended for pancreatic cancer in 2019, but it was not recommended for lung cancer due to the high risk of bleeding (Farge et al., 2019). Finding a drug for thromboprophylaxis without the risk of bleeding remains a challenge for NSCLC patients.

Aspirin, a widely recognized nonselective cyclooxygenase (COX) inhibitor, was initially used as a pain killer before its use extended to the treatment of cardiovascular diseases, with dose adjustments made due to its antiinflammatory effect (Menter and Bresalier, 2023). Inflammation plays a significant role in cancerous tumor formation, and its mitigation could potentially diminish apoptotic and angiogenic cellular events (Menter and Bresalier, 2023). Prostaglandin E2 (PGE2) is a major instigator of inflammation and carcinogenesis, and aspirin has been shown to effectively reduce its levels (Menter and Bresalier, 2023). Lastly, aspirin has been repurposed for colorectal cancer, but its biological effects are not yet fully understood, and ongoing investigations are examining its potential efficacy in various other cancer types (Ricciotti and FitzGerald, 2021; Menter and Bresalier, 2023). In one such study, after NSCLC resection, 75 mg of aspirin was administered to decrease the mortality rate (NCT01058902). In another study, 350 mg of aspirin was administered to stage IIIb-IV or recurrent NSCLC patients to determine PGE2 biosynthesis inhibition (NCT01707823), but the study results are not posted.

Investigating signaling pathways in drug development for NSCLC provides an understanding of drug resistance mechanisms and helps to overcome the problems. Osimertinib, a third-generation EGFR-TKI, has developed resistance mechanisms through RET fusions (Piotrowska et al., 2018). This resistance can be overcome by reducing the phosphorylation of AKT and extracellular-signal-regulated kinase (ERK) (Piotrowska et al., 2018). Since aspirin reduces AKT phosphorylation, combination therapy of osimertinib and acetylsalicylic is under clinical trial (NCT03532698).

In another phase 2/3 clinical trial, a combinational therapy comprising EGFR-TKI (gefitinib), NSAID (acetylsalicylic acid), and a morning sickness drug (thalidomide) is being investigated to assess the impact of thalidomide on interleukin 2, but the results are not posted (NCT02387086). A combinational therapy of osimertinib with itraconazole (ITR) (NCT02157883) has also been suggested for NSCLC to overcome EGFR-TKI resistance (Vishwanathan et al., 2018). ITR is repurposed for several cancers including prostate, breast, triple-negative breast, ovarian, pancreatic, and lung cancer because of its anticancer properties (Antonarakis et al., 2013; Tsubamoto et al., 2015; Alhakamy and Md, 2019; Wu et al., 2022; Sinsuwan and Norchai, 2023). Besides hedgehog pathway inhibition, ITR’s effect on multiple antiangiogenic pathways was reported in an early phase 1 NSCLC study (NCT02357836) (Gerber et al., 2020). The study also reports a change in energy metabolism via the TCA cycle and requirement for further studies (Gerber et al., 2020). In a phase 2 study (NCT00769600) conducted with 23 NSCLC patients, the combination of ITR with the chemotherapy agent pemetrexed resulted in a statistically significant overall survival rate of 32 months (Rudin et al., 2013). In another phase 2 study (NCT03664115), 60 NSCLC patients showed favorable outcomes following itraconazole treatment combined with chemotherapy, but the difference in overall survival rates was not deemed statistically significant (Mohamed et al., 2021). An alcohol addiction drug, disulfiram, has also been investigated in combination with chemotherapy in NSCLC patients, yielding a significantly high overall survival rate of 7.1 months (NCT00312819) (Nechushtan et al., 2015).

## 4. Nanoparticle-based drug delivery systems for oncology drugs

Nanotechnology is an application of nanoscience that provides unique properties to materials through fabrication and characterization on the nanoscale (Bayda et al., 2019). It encompasses areas from physics, chemistry, and biology to material science, computer science, engineering, and health sciences (Bayda et al., 2019). The utilization of nanotechnology in health sciences is termed nanomedicine, encompassing imaging, detection, and therapeutic drugs (Bayda et al., 2019).

Within nanomedicine, cancer nanomedicine attracts attention because of the advances in cancer therapeutics facilitated through NP-based delivery systems (Bayda et al., 2019). NP-based drug delivery systems enhance drugs by prolonging drug circulation, reducing toxicity, enabling controlled and targeted drug delivery, and increasing the solubility of hydrophobic drugs (Dang and Guan, 2020).

**Figure 3:**
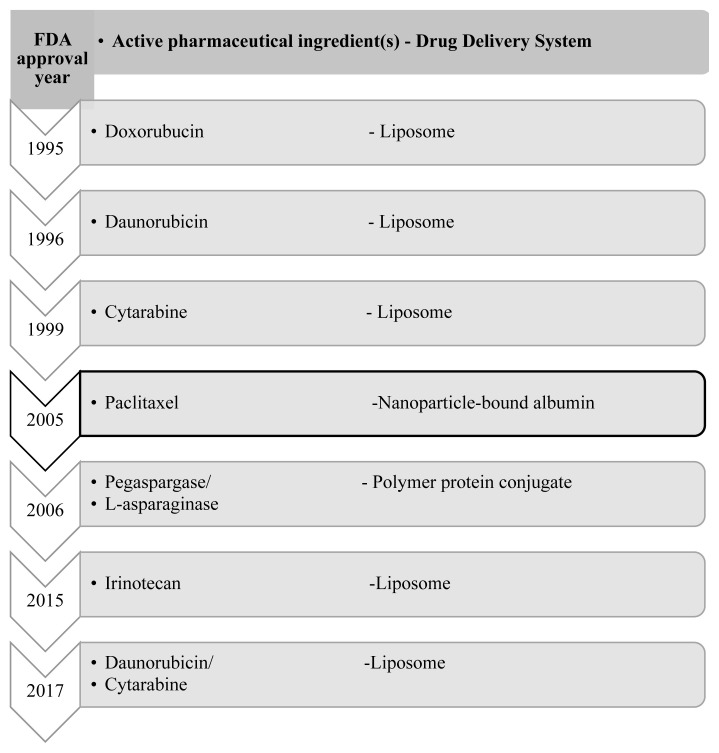
Timeline for NP-based drug delivery systems for cancer treatment. (The NP for NSCLC is indicated with bold line).

Nanoparticles used in cancer treatments can be categorized as liposomes, dendrimers, polymeric nanoparticles, bionanoparticles including viral nanoparticles, protein-based nanoparticles, and apoferritin, inorganic nanoparticles including gold nanoparticles, iron oxide nanoparticles, and silver nanoparticles, other nanoparticles including silica and nanodiamonds (Mukherjee et al., 2019; Mohtar et al., 2021; Fan et al., 2023a). Among these nanomaterials, only liposomes, PEG-PLA polymeric micelle, and nanoparticle-bound albumin were clinically approved for cancer treatments (Foulkes et al., 2020) (Figure 3).

The first FDA-approved NP-based drug was a liposomal doxorubicin commercialized as Doxil in 1995 (Patel, 1996). In Doxil, the pharmacokinetic profile of doxorubicin was enhanced by the liposomal drug delivery system which passively targets the tumor site via enhanced permeability and retention effect (EPR) because of its surface-grafted polyethylene glycol (PEG) chains, thereby prolonging drug circulation and providing longer vascular permeability (Barenholz, 2012). Doxil was used for treating metastatic breast cancer, Kaposi’s sarcoma, multiple myeloma, and ovarian cancer (Barenholz, 2012). However, PEG on the surface of the liposome avoids the drugs from the reticuloendothelial system (RES) and cause drug accumulation in the tissues like the liver, spleen, and bone marrow (Barenholz, 2012). Since then, liposomes have been used as drug carriers for daunorubicin, cytrabine, cytarabine/daunorubicin, irinotecan, vincristine, mifamurtide MTP-PE, and doxorubicin (Giri et al., 2023).

Liposomes enhance the pharmacokinetics and biodistribution of drugs, prolong circulation time, reduce toxicity, and allow passive or active targeted therapy (Milano et al., 2022). Daunorubicin (DaunoXome; Gilead Sciences), cytarabine (DepoCyt; Pacira Pharmaceuticals), and a fixed combination of these drugs in liposome (Vyxeos; Celator/Jazz Pharma) were approved by FDA in 1996, 1999, and 2017, respectively. Daunorubicin (DaunoXome; Gilead Sciences) was approved for Kaposi’s sarcoma. Delayed uptake of liposomes by RES was achieved using a daunoXomes composition of distearoyl phosphatidylcholine and cholesterol in a 2:1 ratio (Petre and Dittmer, 2007). Vyxeos (also known as CPX-351; Celator/Jazz Pharma) is the most recently approved NP-based cancer drug, approved in 2017 (Krauss et al., 2019). Vyxeos liposomal formulations have been introduced for the treatment of acute myeloid leukemia treatments (Krauss et al., 2019). Distearoyl phosphatidylcholine, distearoyl phosphatidylglycerol, and cholesterol are the compositions of the lipid membrane that encapsulates water-soluble drugs daunorubicin and cytarabine (Krauss et al., 2019). Liposome encapsulation not only provides sustained release in 24 h but also preserves drug/drug ratio (Krauss et al., 2019; Tardi et al., 2009). After encapsulation, the terminal half-life of free daunorubicin (18.5 h) and free cytarabine (10 h) was considered nearly similar (Krauss et al., 2019; Tardi et al., 2009). In 2012, liposomal vincristine (Marqibo; Talon Therapeutics/Spectrum Pharmaceuticals) was approved for the treatment of Philadelphia chromosome-negative acute lymphoblastic leukemia; however, it was withdrawn in 2022 due to the lack of clinical benefit from postmarketing clinical trials (Silverman and Deitcher, 2013)^7^. Onivyde (also known as MM-398 or PEP02; Merrimack Pharma) is a liposomal formulation containing irinotecan used in the treatment of metastatic pancreatic cancer (Zhang, 2016). The rapid metabolism of irinotecan results in acute toxicity, but a liposomal formulation overcomes this problem by extending the circulation time of irinotecan (Milano et al., 2022). In addition to general pharmacokinetic improvement, liposomal encapsulation increases the tumor accumulation of irinotecan through the EPR effect (Milano et al., 2022).

A polymer-protein conjugate (Oncaspar; Enzon Pharmaceuticals) was approved in 2006 for acute lymphoblastic leukemia (Dinndorf et al., 2007). In this NP-based drug delivery system, the covalent conjugation of polyethylene glycol to L-asparaginase provides prolonged circulation time, resulting in reduced immunogenicity, which is the main drawback of free asparaginase (Dinndorf et al., 2007).

Commercially available FDA-approved microtubule inhibitor named paclitaxel albumin-stabilized nanoparticle formulation (Abraxane; Abraxis/Celgene) is used for treating metastatic breast cancer, adenocarcinoma of the pancreas, and NSCLC (Attwood, 2012; Attwood, 2013). Noncovalent hydrophobic interactions between human serum albumin-bound paclitaxel conjugates generate nanoparticles with a diameter of approximately 130 nm. This formulation reduces the toxicity (Desai, 2016). In a phase III trial for NSCLC, the overall response rate was 33%, which is considered significant. This therapy is now established as a first-line treatment option in combination with carboplatin for advanced or metastatic NSCLC (Socinski et al., 2012; Desai, 2016).

## 5. Nanoparticle-based drug delivery systems for NSCLC

NP-based drug delivery offers unique advantages for NSCLC therapies compared to free drugs by allowing for the use of different administration routes, generating different cellular responses via material adjustments, and enhancing drug efficacy (Babu et al., 2013; Mukherjee et al., 2019). Nanotechnology-based drug delivery approaches in lung cancer treatment can be expanded to encompass diverse administration routes, including oral, intravenous, and inhalation (Babu et al., 2013). For NSCLC therapeutics, polymeric nanoparticles are more suitable for systemic administration of the drugs and they are also studied for inhalation (Mukherjee et al., 2019). The inhalation route allows delivering higher drug concentrations to the lungs and lower drug concentrations systemically, thereby providing a more localized therapy, with minimized adverse effects (Mukherjee et al., 2019).

Inhaled biopersistent nanoparticles and microparticles have different physicochemical properties (Kreyling et al., 2013). These properties can cause translocation in the circulatory system, and conjugation of nanoparticles with proteins could be a solution for this problem (Kreyling et al., 2013). NP-based drug delivery systems can reduce drugs side effects caused by systemic administration via intravenous routes (Mohtar et al., 2021). It also combats undesirable exhalation of low-inertia nanoparticles which is caused by simple pulmonary delivery of therapeutics (Sung et al., 2007).

A phase I study of DOTAP: Cholesterol-Fus1 Liposome Complex (DOTAP: Chol-fus1) (NCT00059605), a phase I-II study, DOTAP: Chol-TUSC2 (NCT01455389) combination with erlotinib, dexamethasone, and diphenhydramine, a phase I–II study of Manganese Superoxide Dismutase (MnSOD) Plasmid Liposome (NCT00618917) in combination with carboplatin, paclitaxel, and radiotherapy, and a phase I study of lurtotecan liposome (NCT00006036) combined with cisplatin are some of the examples of ongoing clinical trials exploring liposomal therapies for NSCLC. A silica core with an Au (gold) nanoshell, AuroLase, was administered to patients with primary and/or metastatic lung tumors via systemic intravenous infusion and activated at the target site externally with laser radiation delivered by optical fiber via bronchoscopy (NCT01679470). However, this approach is still under development (Singh et al., 2018). The delivery of therapeutic drugs via NP-based drug delivery systems for oncology drugs has exhibited promising efficacy in NSCLC treatment, aiming to regulate the growth of tumor cells (Sharma et al., 2019).

## 6. In vitro studies for repurposed drugs in nanoparticle-based drug delivery systems for NSCLC

The combination of both drug repurposing and NP-based drug delivery systems as a treatment strategy against NSCLC has gained growing interest recently. This combined strategy presents a solution to address the challenges associated with repurposed NSCLC drugs encountered in clinical applications, such as high toxicity, low efficiency, poor solubility, poor metabolism, and complex scaling-up requirements (Najlah et al., 2017; Alhakamy and Md, 2019; Parvathaneni et al., 2020a; Parvathaneni et al., 2020b). FDA-approved drugs repurposed and enhanced with a nanoparticle system for NSCLC in the literature with in vitro studies are listed in Table 4 (Najlah et al., 2017; Butcher et al., 2018; Alhakamy and Md, 2019; Alfaifi et al., 2020; Parvathaneni et al., 2020a; Parvathaneni et al., 2020b; Liu et al., 2023). Among them, disulfiram, nelfinavir, itraconazole, and metformin were involved in clinical trials of repurposing for NSCLC (reported in Section 3), but they were not encapsulated in NP-based drug delivery systems.

### 6.1. Disulfiram: disulfiram-loaded PLGA nanoparticles

Antialcoholism drug disulfiram is metabolized in serum within 4 min which is sufficient for its antialcoholism efficacy (Najlah et al., 2017). However, rapidly metabolized disulfiram becomes a challenge for the drug’s anticancer effect and this can be prevented by PLGA nanoparticles (Najlah et al., 2017). For a better anticancer effect, a copper (Cu)-dependent reaction should take place in the target tumor environment rather than in the bloodstream (Cen et al., 2004).

Disulfiram shows anticancer activity through Cu dependence on its thiuram structures (Butcher et al., 2018). Thiol groups of disulfiram should be chelated with Cu in a tumor environment to act as an anticancer agent via ROS generation-induced apoptosis. However, their chelation is blocked when the drug metabolizes orally, which this limits its anticancer applications (Butcher et al., 2018). Therefore, there is a need for an NP-based delivery system to protect the thiol groups of the drug for the treatment of NSCLC (Butcher et al., 2018).

For better penetration of the drug into the cancer tissue with the EPR effect, disulfiram PLGA nanoparticles require smaller particle sizes with larger surface area. These parameters can be applied during the emulsion-solvent evaporation method (Najlah et al., 2017). PLGA encapsulation enhances the drug’s half-life from 3 min to 60 min (Najlah et al., 2017).

### 6.2. Itraconazole: itraconazole-loaded chitosan-coated PLGA nanoparticles

Poor solubility is a challenge for the antifungal drug ITR. For the treatment of NSCLC, ITR was encapsulated into PLGA nanoparticles via single-emulsion ultrasonication method and then coated with chitosan to overcome the solubility problem (Alhakamy and Md, 2019). The addition of chitosan enhances membrane permeability, while PLGA improves solubility. Overall, this formulation induces apoptosis and is suggested as an anticancer drug (Alhakamy and Md, 2019).

### 6.3. Nelfinavir: nelfinavir-loaded PLGA nanoparticles

Repurposing HIV protease inhibitor nelfinavir exhibited promising results for treating NSCLC; however, its use is limited by the toxicity associated with the required drug dosage (Parvathaneni et al., 2020a). Nelfinavir-PLGA nanoparticles were developed, with a size of 191.1 ± 10 nm, to enhance nelfinavir accumulation in cells while using lower doses (Parvathaneni et al., 2020a). The IC_50_ value of the PLGA-encapsulated drug decreased to 8.3 ± 0.4 μM compared to the free drug, which had an IC_50_ value of 18.1 ± 2.6 μM (Parvathaneni et al., 2020a).

### 6.4. Febuxostat: febuxostat-loaded PEG-coated PLGA nanoparticles

A xanthine oxidase inhibitor named febuxostat is utilized for the treatment of gout patients’ hyperuricemia (Edwards, 2009). Febuxostat-loaded PEG-coated PLGA nanoparticles were synthesized with the nanoprecipitation method because of the poor water solubility of febuxostat (Alfaifi et al., 2020). After nanoparticle synthesis, the IC_50_ value of the febuxostat-loaded nanoparticles decreased to 52.62 ± 2.52 μg/mL compared to the free drug, which had an IC_50_ value of 68 ± 4.12 μg/mL (Alfaifi et al., 2020).

### 6.5. Amodiaquine: amodiaquine-loaded PLGA nanoparticles

Antimalarial drug hydroxychloroquine has been repurposed in a clinical trial for NSCLC (Section 3, NCT01026844, NCT04735068). Another antimalarial drug amodiaquine is a chloroquine analog with a similar mechanism of action except p53 stabilization (Espinoza et al., 2020). Amodiaquine has become a stronger anticancer candidate with an improved understanding of its mechanisms (Espinoza et al., 2020). The drug is considered an inhalation therapy against NSCLC, and it is delivered using amodiaquine-loaded PLGA nanoparticles (Parvathaneni et al., 2020b). The high-pressure homogenization method is used to increase the scale-up feasibility of nanoparticle systems and ensure reproducible product quality for the production of inhalable nanoparticulate systems (Parvathaneni et al., 2020b).

### 6.6. Sertaconazole: hyaluronic acid-TPGS-sertaconazole nanoparticles

Although the antifungal agent sertaconazole is not under clinical investigation for NSCLC, its repositioning potential has been reported, and the molecular mechanism of NSCLC cells has been revealed (Zhang et al., 2021). Sertaconazole triggers proapoptotic autophagy by preventing ubiquitination-mediated proteasomal degradation of TNF receptor type 1 associated death domain protein (TRADD) (Zhang et al., 2021). Moreover, microtubule depolymerization and binding of tubulin induced by sertaconazole cause toxicity in HeLa cells, which suggests the drug may have potential as an anticancer agent, warranting further studies (Sebastian and Rathinasamy, 2021). To reduce toxicity and increase the efficacy of sertaconazole, the thin film dispersion method was used in conjunction with hyaluronic acid and D-α-tocopheryl polyethylene glycol 1000 succinate (TPGS) to provide CD44-specific, pH-responsive delivery, better solubility, and accumulation at the tumor site (Liu et al., 2023). To the best of our knowledge, this study has been the most recently reported combination strategy of both drug repurposing and NP-based drug delivery systems as a treatment against NSCLC (Liu et al., 2023).

## 7. Other nanotechnology-based therapies for NSCLC: vaccines

Cancer vaccines can be categorized into DNA, RNA, peptide, cell-based viral and bacterial vector vaccines (Fan et al., 2023b). These vaccines are being developed to overcome the primary challenge of generating antigen-specific T cell responses in cancer vaccines (Fan et al., 2023b). The application of mRNA vaccines has been accelerated by the COVID-19 pandemic. It’s anticipated that the authorization of personalized mRNA vaccines for cancer will occur by 2030 (Fan et al., 2023b). However, in addition to mRNA vaccine challenges, DNA, peptide, cell-based viral and bacterial vector vaccines also have challenges that need to be addressed (Fan et al., 2023b). To date, there is no available approved vaccine for lung cancer. However, with the increasing understanding of immunotherapies and advancements in nanotechnology, clinical trials have been conducted on several vaccine types including peptide vaccines. These clinical trials comprise vaccines against lung cancer, including peptide-based vaccines such as the MUC1 peptide-Poly-ICLC vaccine (NCT03300817, NCT01720836) and HLA-A*2402restricted URLC10, CDCA1, and KIF20A peptides vaccine (NCT01069575).

## 6. Conclusion and future perspectives

This review summarizes the current knowledge regarding NSCLC treatments, including existing approved drugs, drug repurposing with details of clinical study advances, and NP-based drug delivery systems. Additionally, it discusses the molecular aspects of the recently approved NSCLC drug, the ROS1 TKI repotrectinib, which was authorized in November 2023, alongside all other approved NSCLC drugs. However, resistance mechanisms in the NSCLC microenvironment occurs rapidly, primarily affecting detectable specific genes like EGFR and ALK (Liu et al., 2020), which necessitates personalized medicine approaches. The effectiveness of personalized medicine is high because of various genomic alterations in NSCLC as in the example of KRAS G12 (Jacobs et al., 2021). These specific genes serve as excellent targets for personalized medicine in NSCLC, highlighting a growing need for targeted therapies. While cancer nanomedicine enables targeted therapy, one of the main challenges is achieving drug release at the target area with the intended dose and timing (Fan et al., 2023a).

High tumor accumulation, accurate subcellular localization, and efficient cellular internalization are additional obstacles in cancer nanomedicine (Fan et al., 2023a). Even though cellular internalization efficiency can be enhanced by targeted therapy, determining ligands without eliciting toxicity and immune responses and releasing only at the target cell remains challenging (Fan et al., 2023a). Further improvements in cancer nanomedicine could provide better solutions. In vitro studies of both drug repurposing and NP-based drug delivery systems as a treatment strategy against NSCLC are studied and further clinical experiments are required. Advances in NP-based drug delivery systems provide a promising resource for treatment options gained by the drug repurposing approach. The drug repurposing approaches are also improved with a new area of interest, artificial intelligence (AI), which is a rapidly growing technology area that can provide extensive information to target tumors’ molecular profile and their microenvironment to predict expected adverse events, acquired or inherent resistance mechanisms, biomarkers, and the choice of combination therapy for personalized medicine. AI has been applied in oncology, specifically, in radiotherapy and immune-oncology (El Naqa et al., 2023). These preapplications indicate that AI is a potential candidate bridging the gaps in detection, diagnosis, and therapeutics.

## Figures and Tables

**Table 1:** NIH-approved drug list for NSCLC (Cho et al., 2023; NIH, 2023^1^).

Main class	Classes of active pharmaceutical ingredient (s)	Name of the active pharmaceutical ingredient (s)	Commercial name of the drug/company	Initial year of approval/approval for NSCLC	References
**Tyrosine kinase inhibitors (TKIs)**	ROS1 TKI	**Repotrectinib**	Augtyro, Bristol-Myers Squibb Company	2023	**(Cho et al., 2023)** **(FDA, 2023)** 2
	ALK inhibitor	**Ceritinib**	ZYKADIA Novartis Pharmaceuticals Corporation	2014/2014	**(Dhillon and Clark, 2014)**
	ALK inhibitor, a small-molecule TKI that inhibits the activity of the ALK fusion proteins, MET, ROS1, and MST1R (RON)	**Crizotinib**	XALKORI Pfizer Laboratories Div Pfizer Inc.	2011	**(Kazandjian et al., 2014)**
	Kinase inhibitor	**Alectinib**	ALECENSA Genentech, Inc.	2015/2015	**(Larkins et al., 2016)**
	ALK inhibitor	**Brigatinib (AP26113)**	ALUNBRIG Takeda Pharmaceuticals America, Inc.	2017/2017	**(Markham, 2017)**
	BRAF inhibitor	**Dabrafenib**	TAFINLAR Novartis Pharmaceuticals Corporation	2013/2017	**(Odogwu et al., 2018)**
	MET TKI	**Tepotinib**	TEPMETKO EMD Serono, Inc.	2021/2021	**(Mathieu et al., 2022)**
	MET TKI	**Capmatinib hydrochloride**	TABRECTA Novartis Pharmaceuticals Corporation	2020/2020	**(Mathieu et al., 2022)**
	RET receptor TKI	**Pralsetinib**	GAVRETO Blueprint Medicines Corporation	2020/2020	**(Griesinger et al., 2022)**
	RET kinase inhibitor	**Selpercatinib**	RETEVMO Eli Lilly and Company	2020/2020	**(Bradford et al., 2021)**
	Mitogen-activated extracellular signal-regulated kinase 1 (MEK1) and MEK2 inhibitor	**Trametinib**	MEKINIST Novartis Pharmaceuticals Corporation	2013/2017	**(Odogwu et al., 2018)**
	An irreversible inhibitor of the ErbB family of tyrosine kinases, inhibits EGFR (ErbB1), HER2 (ErbB2), and HER4 (ErbB4)	**Afatinib dimaleat**	GILOTRIF Boehringer Ingelheim Pharmaceuticals, Inc.	2003/2013	**(Dungo and Keating, 2013)**
	EGFR TKI	**Osimertinib**	TAGRISSO (AZD9291) AstraZeneca Pharmaceuticals LP	2015/2015	**(Greig, 2016)**
	ALK inhibitor	**Lorlatinib**	LORBRENA Pfizer Laboratories Div Pfizer Inc.	2018/2018	**(Syed, 2019)**
	ALK inhibitor	**Entrectinib**	ROZLYTREK Genentech, Inc.	2019/2019	**(Marcus et al., 2021)**
	EGFR TKI	**Erlotinib**	TARCEVA Genentech, Inc.	2004/2004	**(Cohen et al., 2005)**
	EGFR TKI	**Gefitinib**	IRESSA AstraZeneca Pharmaceuticals LP	2003/2003–2015	**(Cohen et al. 2004) (Kazandjian et al. 2016)**
	EGFR inhibitor	**Mobocertinib**	Exkivity Takeda Pharmaceuticals USA, Inc.	2021/2021	**(Markham, 2021)**
	Second-generation TKI	**Dacomitinib**	VIZIMPRO Pfizer Laboratories Div Pfizer Inc.	2018/2018	**(Shirley, 2018)**
**KRAS inhibitors**	Inhibitor of the RAS GTPase family	**Adagrasib (**MRTX849**)**	KRAZATI Mirati Therapeutics, Inc.	2022/2022	**(Dhillon, 2023)**
	Inhibitor of the RAS GTPase family	**Sotorasib (**AMG-510**)**	LUMAKRAS Amgen Inc.	2021/2021	**(Blair, 2021)**
**Antibodies**	Cytotoxic T-lymphocyte-associated antigen 4 (CTLA-4) blocking antibody	**Tremelimumab**	IMJUDO AstraZeneca Pharmaceuticals LP	2022/2022	**(Keam, 2023)**
	Bispecific EGF receptor-directed and MET receptor-directed antibody	**Amivantamab-vmjb**	RYBREVANT Janssen Biotech, Inc.	2021/2021	**(Chon et al., 2023)**
	A humanized monoclonal that binds directly to PD-L1, blocking its interactions with the PD-1 and CD80 molecule receptors.	**Atezolizumab**	TECENTRIQ Genentech, Inc.	2016/2016	**(Weinstock et al., 2017)**
	Recombinant humanized monoclonal IgG_1_ antibody, Vascular endothelial growth factor (VEGF) inhibitor	**Bevacizumab**	AVASTIN Genentech, Inc.	2004/2006	**(Cohen et al., 2007)**
	Recombinant humanized monoclonal IgG_1_ antibody, VEGF inhibitor	**Bevacizumab**	(MVASI, Amgen Inc), (ZIRABEV, Pfizer Laboratories Div Pfizer Inc), (VEGZELMA- CELLTRION USA, Inc.), (ALYMSYS- Amneal Pharmaceuticals LLC) Biosimilar to avastin	2017,2019, 2022,2022	**(Casak et al., 2018)**
	IgG monoclonal antibody directed against the EGFR	**Necitumumab**	PORTRAZZA Eli Lilly and Company	2015/2016	**(Garnock-Jones, 2016)**
	CTLA-4 blocking antibody	**Ipilimumab**	YERVOY E.R. Squibb & Sons, L.L.C.	2011/2020	**(Vellanki et al., 2021)**
	Programmed death receptor-1 (PD-1)-blocking antibody	**Nivolumab**	OPDIVO E.R. Squibb & Sons, L.L.C.	2014/2020	**(Vellanki et al., 2021)**
	HER2-directed antibody and topoisomerase inhibitor conjugate	**Trastuzumab deruxtecan**	ENHERTU Daiichi Sankyo, Inc., AstraZeneca	2019/2019	**(Keam, 2020)**
	PD-1 monoclonal antibody	**Cemiplimab-rwlc**	LIBTAYO- Regeneron Pharmaceuticals, Inc.	2018/2021	**(Akinboro et al., 2022)**
	PD-1 monoclonal antibody	**Pembrolizumab**		2016	**(Akinboro et al., 2022)**
**Others**	A third-generation vinca alkaloid	**Vinorelbine tartrate**	NAVELBINE Pierre Fabre Pharmaceuticals, Inc.	1994/1994	**(Toso and Lindley, 1995)**
	Antineoplastic agent	**Methotrexate sodium**	TREXALL Teva Women’s Health, Inc.	1953	**(TREXALL, 1953)** 3
	Antineoplastic agent	**Methotrexate**	XATMEP Azurity Pharmaceuticals, Inc.	1953	**(XATMEP, 1953)** 4
	Folate analog metabolic inhibitor	**Pemetrexed disodium**	ALIMTA Eli Lilly and Company	2004	**(Cohen et al., 2005)**
	Microtubule inhibitor	**Paclitaxel**	ABRAXANE Abraxis BioScience, LLC	2005/2012	**(Gradishar, 2006; Hirsh, 2014)**
	Direct VEGF receptor 2 antagonist	**Ramucirumab**	CYRAMZA Eli Lilly and Company	2014/2014	**(Larkins et al., 2015)**
	Taxoid antineoplastic agent microtubule inhibitor	**Docetaxel**	Taxotere Aventis Pharma	1996/2002	**(Davies et al., 2003)**
	Mammalian target of rapamycin (mTOR) inhibitor	**Everolimus**	Afinitor, Novartis	2009/2016	**(FDA, 2016)** 5
	Nucleoside metabolic inhibitor a deoxycytidine analog	**Gemcitabine**	Gemzar Infugem	1996	**(Natale, 2005)**

**Table 2:** FDA-approved active substances undergoing repurposing for NSCLC in clinical trials (clinicaltrials.gov)^6^

Active pharmaceutical ingredient (s)	First indication	Action mechanism	Clinical trials.gov(clinicaltrials.gov) 3 Identifier	Study phase
**Nadroparin**	Prophylaxis of thrombotic events, non-Q-wave myocardial infarction, deep vein thrombosis, and unstable angina.	Heparin	NCT01980849	3
**Statin**, Multiple cardiovascular drugs related to the “Golden Triangle”	Cardiovascular diseases	HMG-CoA (hydroxymethylglutaryl-coenzyme A) reductase inhibitor	NCT04980716	3
**Aspirin (acetylsalicylic acid)**	Pain, myocardial infarction (MI)	Nonselective cyclooxygenase (COX) inhibitor	NCT01707823	3
			NCT01058902	3
**Osimertinib and aspirin (acetylsalicylic acid)**	**Osimertinib**: Nonsmall-cell lung carcinoma. **Aspirin (Acetylsalicylic acid):** Pain, myocardial infarction (MI)	**Osimertinib**: tyrosine kinase inhibitor **Aspirin (Acetylsalicylic acid):** nonselective cyclooxygenase (COX) inhibitor	NCT03532698	Observational
**Thalidomide**	Morning sickness, erythema nodosum leprosum, multiple myeloma	Immunosuppressive and antiangiogenic activity	NCT02387086	2/3
**Disulfiram**	Alcohol addiction	Carbamate derivative	NCT00312819	2/3
**Itraconazole**	Fungal infections	Antifungal agent, cytochrome P450 14α-demethylase inhibitor, CYP3A4 inhibitor	NCT02357836	1
			NCT00769600	2
**Itraconazole + osimertinib**	Fungal infections	Antifungal agent, cytochrome P450 14α-demethylase inhibitor, CYP3A4 inhibitor	NCT02157883	1
Itraconazole +chemo	Fungal infections	antifungal agent, cytochrome P450 14α-demethylase inhibitor, CYP3A4 inhibitor	NCT03664115	2
**Nelfinavir**	HIV infection	HIV-1 protease inhibitor	NCT01447589	1/2
**Nelfinavir + proton therapy**	HIV infection	HIV-1 protease inhibitor	NCT01108666	2
**Nelfinavir** in combination with radiotherapy and chemotherapy	HIV infection	HIV-1 protease inhibitor	NCT00791336	2
**Valproic Acid**	Complex partial seizures	Anticonvulsant	NCT01203735	1/2
**Decitabine + Valproic acid**	**Decitabine**: myelodysplastic syndromes **Valproic acid**: complex partial seizures	**Decitabine**: chemotherapeutic pyrimidine nucleoside analogue **Valproic acid**: anticonvulsant	NCT00084981	1
**Metformin**	Type 2 diabetes mellitus	Biguanide antihyperglycemic	NCT03086733	2
			NCT03071705	
			NCT04170959	2
			NCT02186847	2
			NCT01864681	2
			NCT02019979	2
**Metformin in combination with radiation therapy**	Type 2 diabetes mellitus	Biguanide antihyperglycemic	NCT02285855	2
**Rosuvastatin + erlotinib**	**Rosuvastatin:** cardiovascular disease; myocardial infarction and stroke **Erlotinib**: pancreatic cancer, NSCLC	**Rosuvastatin:** HMG-CoA reductase inhibitor **Erlotinib**: EGFR TKI	NCT00966472	1
**Erlotinib + hydroxychloroquine**	**Erlotinib**: pancreatic cancer, NSCLC **Hydroxychloroquine:** malaria, lupus, rheumatoid arthritis	**Erlotinib:** EGFR TKI **Hydroxychloroquine:** antimalarial, disease-modifying anti-rheumatic drug (DMARD)	NCT01026844	1
**Binimetinib + hydroxychloroquine**	**Binimetinib:** metastatic melanoma **Hydroxychloroquine:** malaria, lupus, rheumatoid arthritis	**Binimetinib:** elective oral MEK ½ inhibitor **Hydroxychloroquine:** antimalarial, DMARD	NCT04735068	2
**Steroid therapy, such as dexamethasone**	**Dexamethasone**: bronchial asthma, endocrine and rheumatic disorders	**Dexamethasone**: glucocorticoid	NCT00403065	1
**Midazolam** + **TAK**-**788** (Mobocertinib)	**Midazolam**: seizures, anesthesia, and anxiety disorders **TAK**-**788** (mobocertinib): NSCLC (EGFR exon 20 insertions)	**Midazolam**: a short-acting benzodiazepine, hypnotic-sedative **TAK**-**788** (mobocertinib): TKI	NCT04051827	1
**Zoledronic acid**	Hypercalcemia of malignancy, multiple myeloma, prostate cancer after hormonal therapy and bone metastasis from solid tumors	Nitrogen-containing bisphosphonate	NCT00099541 (completed)	4
**Captopril**	Hypertension, heart failure	Angiotensin-converting enzyme (ACE) inhibitor	NCT00077064	2

6 ClinicalTrials.gov Website: https://clinicaltrials.gov. [accessed 14 12 2023]

**Table 3:** Repurposed active substances’ on clinical trials for NSCLC (clinicaltrials.gov) 6.

Active pharmaceutical ingredient (s)	First indication	Action mechanism	Clinical trials.gov (clinicaltrials.gov)^6^ Identifier	Study phase
**Beta-glucan- Imucell WGP**	Dietary supplement **Not FDA approved**	Carbohydrate	NCT00682032	Not applicable
**Marimastat**	Cancer **Not FDA approved**	Antineoplastic drug, matrix metalloprotease inhibitor	NCT00002911	3
A**ntroquinonol**	acute myeloid leukemia **Not FDA approved**	diterpenoid	NCT02047344	2
**L-alanosine**	**Not FDA approved**	Antibiotic	NCT00062283	2
**Isoquercetin**	Kidney cancer, renal cell carcinoma, advanced renal cell carcinoma, thromboembolism of vein in pancreatic cancer, and thromboembolism of vein VTE in colorectal cancer **Not FDA approved**	Dietary ingredient	NCT02195232	2/3
**Vitamin A**	Vitamin A deficiency	Retinol	NCT03870529	1
**Melatonin**	Non-24-hour sleep-wake disorder in blind individuals without light perception **FDA orphan drug**	Endogenous hormone	NCT00668707	3

6 clinicaltrials.gov Website: https://clinicaltrials.gov. [accessed 14 12 2023]

**Table 4:** In vitro studies of repurposed drugs in NP-based systems for NSCLC.

Active pharmaceutical ingredient (s)	First indication	Nanoparticle system	Method to synthesize nanoparticle	NSCLC cell line	References
**Niclosamide**	Antihelmintic	Pluronic1 P123/F127 mixed micelles	Thin-film hydration	A549	(Russo et al. 2016)
**Disulfiram**	Antialcoholism medicine	Drug-loaded PLGA nanoparticles	Emulsion/solvent evaporation	A549	(Najlah et al. 2017)
		Novel soluble copper-DDC compound	The need for a system for novel soluble copper-DDC compounds is emphasized	A549	(Butcher et al. 2018)
**Itraconazole**	A broad-spectrum antifungal	Chitosan-coated PLGA nanoparticles of itraconazole	Single-emulsion ultrasonication	H1299	(Alhakamy and Md 2019)
**Nelfinavir**	antiretroviral	PLGA nanoparticles of nelfinavir	Emulsion/solvent evaporation	A549, H4006, H460	(Parvathaneni et al. 2020a)
**Amodiaquine**	Antimalarial compound (Basoquin)	Amodiaquine-loaded nanoparticles inhalable DoE-based nanoparticles	High-pressure homogenization	A549, H4006, H358, H2122, H460 and H157	(Parvathaneni et al. 2020b)
**Febuxostat**	Xanthine oxidase inhibitor hyperuricemic agent	Febuxostat (FBX)-loaded PEG-coated PLGA nanoparticles	Nanoprecipitation	A549	(Alfaifi et al. 2020)
**Metformin**	Biguanide antihyperglycemic	Sterosomes	Thin-film hydration	A549	(Osama et al. 2020)
**Pirfenidone**	Antifibrotic	Liposome	Thin-film hydration	H4006, A549	(Parvathaneni et al. 2020c)
**Quinacrine**	Antimalarial	Bovine serum albumin-modified cationic nanoparticles	Multiple-emulsion/solvent evaporation	A549	(Vaidya et al. 2020)
**Bedaquiline**	Antitubercular	Cubosome	Single-emulsion/solvent evaporation	A549	(Patil et al. 2021)
**Sertaconazole**	Antifungal	Nanoplatform	Thin-film dispersion	H460, A549	(Liu et al. 2023)

## Footnotes

1 NIH (2023) Drugs approved for non-small cell lung cancer. Website: https://www.cancer.gov/about-cancer/treatment/drugs/lung#1 [accessed 08 09 2023]

2 FDA (2023) FDA approves repotrectinib for ROS1-positive non-small cell lung cancer. Website: https://www.fda.gov/drugs/resources-information-approved-drugs/fda-approves-repotrectinib-ros1-positive-non-small-cell-lung-cancer [accessed: 01 12 2023]

3 TREXALL (1953). Website: https://dailymed.nlm.nih.gov/dailymed/drugInfo.cfm?setid=e942f8db-510f-44d6-acb5-b822196f5e8c [accessed 01 12 2023]

4 XATMEP (1953). Website: https://dailymed.nlm.nih.gov/dailymed/drugInfo.cfm?setid=aec9984e-34c5-481b-b6bf-9bb5caf1daf8 [accessed 01 12 2023]

5 FDA (2016) Everolimus (Afinitor). Website: https://www.fda.gov/drugs/resources-information-approved-drugs/everolimus-afinitor [accessed 01 12 2023]

6 ClinicalTrials.gov Website: https://clinicaltrials.gov. [accessed 14 12 2023]

7 FDA (2022). Acrotech biopharma LLC; withdrawal of approval of new drug application for MARQIBO (VinCRIStine sulfate LIPOSOME injection), 5 milligrams/5 milliliters. Website: https://www.federalregister.gov/d/2022-09235 [accessed 22 11 2023]
